# Multicenter randomized phase III trial of Epirubicin plus Paclitaxel *vs* Epirubicin followed by Paclitaxel in metastatic breast cancer patients: focus on cardiac safety

**DOI:** 10.1038/sj.bjc.6601883

**Published:** 2004-06-01

**Authors:** E Baldini, T Prochilo, B Salvadori, A Bolognesi, D Aldrighetti, M Venturini, R Rosso, F Carnino, L Gallo, P Giannessi, P F Conte, C Orlandini, P Bruzzi

**Affiliations:** 1 Medical Oncology Department, S. Chiara University-Hospital, Via Roma 67, 56132 Pisa, Italy; 2 Medical Oncology Department, S. Raffaele Hospital, 20100 Milano, Italy; 3 Medical Oncology, National Institute for Cancer Research, 16132 Genoa, Italy; 4 Gynecology Department, S. Anna Hospital, 10100 Torino, Italy; 5 Medical Oncology, Galliera Hospital, 16132 Genova, Italy; 6 Medical Oncology Hospital, 57100 Livorno, Italy; 7 Medical Oncology Department, University of Modena e Reggio, 41100 Modena, Italy

**Keywords:** multicenter trial, Epirubicin, Paclitaxel, cardiotoxicity, risk factors

## Abstract

The aim of the study was to evaluate cardiac safety of two different schedules of Epirubicin and Paclitaxel in advanced breast cancer patients enrolled into a multicenter randomized phase III trial. Patients received Epirubicin 90 mg m^−2^ plus Paclitaxel 200 mg m^−2^ (3-h infusion) on day 1 every 3 weeks for eight courses (arm A), or Epirubicin 120 mg m^−2^ on day 1 every 3 weeks for four courses followed by four courses of Paclitaxel 250 mg m^−2^ on day 1 every 3 weeks (arm B). Left ventricular ejection fraction was evaluated by bidimesional echocardiography at baseline, after four and eight courses of chemotherapy and every 4 months during follow-up. Baseline median left ventricular ejection fraction was 60% in arm A and 65% in arm B; after four courses, figures were 57 and 60%, respectively. After eight courses, the median left ventricular ejection fraction in arm A declined to 50% while no further reduction was detected in arm B by adding four courses of high-dose Paclitaxel. Seven episodes of congestive heart failure were observed during treatment in arm A. Present monitoring demonstrated that the risk of congestive heart failure or impairment in the cardiac function correlated only with the cumulative dose of Epirubicin; no impact on cardiotoxicity can be attributed to high-dose Paclitaxel.

Anthracyclines (Doxorubicin and Epirubicin) still represent the most active and widely used cytotoxic drugs in the management of breast cancer: their introduction in the adjuvant setting provided a modest, but significant, improvement in survival and their use in first-line therapy for advanced disease significantly improved response rate and palliation (A’Hern *et al*, 1993; EBCTCG, 1998). However, successful use is limited by their cumulative dose-related cardiotoxicity, less severe after Epirubicin than after equimolar doses of the parental compound, Doxorubicin (Cersosimo and Hong, 1986). In the last decade, the introduction of taxanes (Paclitaxel and Docetaxel) in the treatment of breast cancer have provided additional therapeutic options for patients with advanced disease, and these molecules have been widely tested in combination with anthracyclines yielding promising results. However, together with the high rate of remission, some authors underlined the unexpectedly high incidence of severe cardiotoxic events when the combination Paclitaxel/Doxorubicin was used. In these cases, Paclitaxel seemed to be actively responsible for enhanced cardiotoxicity of the combination since it significantly modified the pharmacokinetics of Doxorubicin and its cardiotoxic metabolites (Gianni *et al*, 1995a, 1995b; Gehl *et al*, 1996). At comparable dose levels, Epirubicin is significantly less cardiotoxic than Doxorubicin (Perez *et al*, 1991) and Paclitaxel seems to play a minor role on Epirubicin metabolism when the two drugs are used in combination (Conte *et al*, 1997, Danesi *et al*, 2002); this data could explain, in part, the more favourable cardiotoxic profile reported for the douplet Paclitaxel/Epirubicin.

More recently, the sequential administration of antiblastic drugs has attracted the attention of researchers and clinicians. This alternative schedule of administration is supported by an interesting preclinical rationale (Norton, 1997); in addition, the sequence of Epirubicin followed by Paclitaxel (3-h infusion) is also supported by clinical data showing the partially non crossresistance of the two drugs (Gianni *et al*, 1995a; Seidman *et al*, 1995). Our group carried out a multicenter randomised phase III trial testing the safety, activity and efficacy of two different schedules of Epirubicin and Paclitaxel (concurrent *vs* sequential) in advanced breast cancer patients. In both the arms, drugs were administered at their maximum tolerated doses (not requiring colony-stimulating factor support) either in combination (Conte *et al*, 1997) or as single-agent (Bashtold *et al*, 1996). The overall results of sequentially administered Epirubicin and Paclitaxel seemed to be comparable to those obtained with the combination of the two drugs (Baldini *et al*, 2002); however, no data, so far exists, in literature on the cardiac safety of this alternative schedule.

The present report concerns the systematical monitoring of cardiac functions performed in 136 patients enrolled into the above-mentioned randomised trial; we evaluated the relationship between schedule, cumulative dose of Epirubicin, type of prior adjuvant treatment and risk of developing a cardiotoxic event.

## PATIENTS AND METHODS

### Patients

From May 1999 to July 2001, 202 breast cancer patients, from seven Institutions (six Italian, one Spanish), were enrolled. Bidimensionally measurable metastatic disease, ECOG performance status ⩽2, normal haematologic (haemoglobin >11 g dl^−1^, wbc>4.000 *μ*l^−1^, platelet count ⩾100.000 *μ*l), hepatic (bilirubin ⩽2 × upper normal limit) and renal (creatinine ⩽2 × upper normal limit) functions were required. Baseline left ventricular ejection fraction (LVEF) greater than 50% at bidimensional ultrasonography and no clinical history of cardiac disorders were mandatory. Prior adjuvant chemotherapy was allowed if stopped at least 1 year before entering the study; adjuvant Epirubicin-based chemotherapy was permitted if the cumulative dose did not exceed 360 mg m^−2^. Patients submitted to prior chemotherapy for advanced disease were not allowed, and only one hormonal therapy (adjuvant or advanced) was permitted.

Out of 202 patients, 136 underwent systematic cardiac monitoring performed during treatment and follow-up.

### Treatment plan

Patients were randomised to:

*Arm A*: Epirubicin 90 mg m^−2^ (bolus i.v.) immediately followed by Paclitaxel 200 mg m^−2^ (3-h infusion) on day 1 every 3 weeks for a maximum of eight courses (E+P).

*Arm B*: Epirubicin (single agent) 120 mg m^−2^ (bolus i.v.) on day 1 every 3 weeks for four courses, followed by four courses of Paclitaxel (single agent) 250 mg m^−2^ on day 1 every 3 weeks (E → P).

### Evaluation of cardiac function

In both arms, clinical examination and ECG were performed at baseline and at every two courses of chemotherapy; LVEF was determined by bidimensional echocardiography on study entry, after four and eight courses of treatment; cardiac monitoring was continued every 4 months during follow-up. Toxicity was graded according to the New York Heart Association (NYHA) criteria for cardiac failure (NYHA, 1964).

### Objective and statistical analysis

The primary end point was the incidence of cardiac events in both arms. A cardiac event was defined as a 20% decline in LVEF from baseline or a congestive heart failure (CHF). The diagnostic criteria for CHF were history of breathlessness, dyspnea, presence of peripheral oedema, cardiomegaly on chest radiography with or without pulmonary congestion and pleural effusion. At the occurrence of clinical symptoms of CHF, a reduced LVEF contributed to diagnosis. The risk of developing a cardiotoxic event as a function of cumulative dose of Epirubicin and schedule was estimated by the Kaplan–Meier method. In order to compare the risk of development of a cardiac event in two groups, the Log-rank test was used. Data were analysed using SPSS/PC+11.5 statistical softwere.

## RESULTS

Out of 136 patients, 72 received Epirubicin plus Paclitaxel (564 total courses) and 64 Epirubicin followed by Paclitaxel (492 total courses). No significant difference in risk factors for heart disease (age, hypertension, left breast irradiation) was observed between the two arms. In both groups, about 25% of patients had prior anthracycline-based adjuvant therapy (Table 1

**Table 1:** Patients characteristics

	**E+P**		**E → P**	
	**No.**	**%**	**No.**	**%**
No. assessable	72		64	
Median age (range)	58 (36–73)		58 (30–73)	
Median ECOG PS	0 (0–1)		0 (0–1)	
Disease status at random				
Metastatic *ab initio*	19	26.4	10	15.7
Relapsed after surgery	53	73.6	54	84.3
Prior adjuvant CT	32	44.4	33	51.5
Epirubicin based	18	25	16	25

PS=perfofmance status; ECOG=European Collaborative Oncology Group; CT=chemotherapy

).

**Figure 1:**
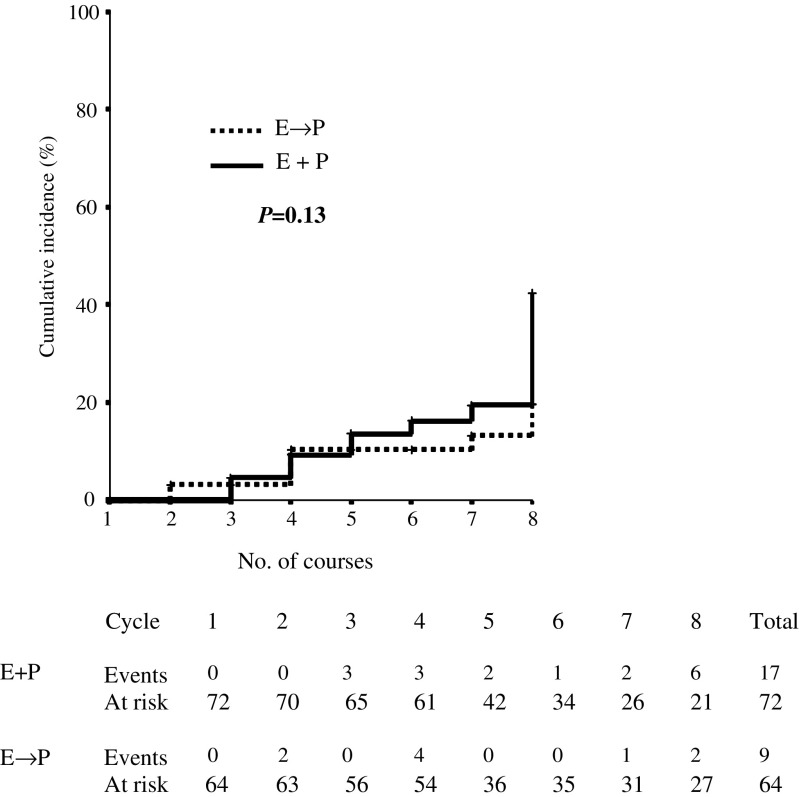
Incidence of cardiac events by treatment arm.

After four courses of treatment, in both arms, a modest decrease in the median LVEF was observed: from 60 to 57% in arm A and from 65 to 60% in arm B; the median delivered dose of Epirubicin was 360 mg m^−2^ (range 90–720 mg m^−2^) and 480 mg m^−2^ (range 240–840 mg m^−2^) in arms A and B, respectively. In arm A, four additional courses of concomitant E+P induced a further drop of median LVEF to 50% (median delivered doses of E 720 mg m^−2^ (range 90–1080 mg m^−2^)). In arm B, no further reduction in LVEF was observed by adding, sequentially, four courses of high-dose Paclitaxel. After four courses of chemotherapy, 12 patients (six for each arm) showed a 20% decline in LVEF (from baseline): at the end of the treatment (eight courses), the same reduction was detected in 17 patients arm A and nine patients arm B (Figure 1

).

No episode of CHFs was observed in arm B. Seven episodes of CHF were reported in arm A: characteristics of the patients are listed in Table 2

**Table 2:** Characteristics of patients with CHF

**E+P**							
**Number of patients**	**7**						
Age (years)	71	67	61	46	56	46	70
PS (ECOG)	1	0	0	0	0	0	0
Prior adjuvant E (360 mg m^−2^)	No	Yes	No	Yes	Yes	Yes	No
Left breast radiotherapy	No	Yes	Yes	Yes	Yes	Yes	Yes
Baseline LVEF (%)	50	58	60	50	55	55	55
CHF class NYHA	IV	III	III	IV	IV	IV	III
E cumulative dose (mg m^−2^)	90	630	720	1080	1080	1080	360
Death due heart failure	Yes	No	No	No	No	No	No

CHF=congestive heart failure; LVEF=left ventricular ejection fraction; NYHA=New York Heart Association. PS=performance status; ECOG=European Collaborative Oncology Group

. Four out of seven patients had prior Epirubicin-based adjuvant therapy. One patient (71 years old, no prior adjuvant anthracycline, no breast radiotherapy) was admitted 10 days after the first course of chemotherapy because of severe dyspnea: she had pulmonary congestion, a LVEF of 25% (baseline 50%), peripheral oedema and oliguria; a grade 3 neutropenia was also observed: she died 2 days after admission. Three patients were admitted a few days after the end of combination therapy (eight total courses), with clinical signs and symptoms of CHF (cumulative dose of Epirubicin 1080 mg m^−2^): a chest X-ray showed cardiomegaly in two out of three patients and their LVEFs dropped to 28 and 30%. They were treated with diuretics, angiotensin converting enzyme (ACE) inhibitors, anti-arrhythmics and anti-coagulants at first; subsequently, they were stabilised with digitalo-diuretics therapy and cardioaspirin. These patients died of disease 16, 24 and 18 months after CHF with persistent severe impairment in the cardiac function. The last three patients presented class III CHF (NYHA): one patient had received adjuvant Epirubicin and three courses of E+P when the symptoms appeared (cumulative dose of Epirubicin 630 mg m^−2^), while the other two were anthracycline-naïve and showing CHF after a cumulative dose of 720 mg m^−2^ (eight total courses) and 360 mg m^−2^ (four total courses). They required admission because of dyspnea but no cardiomegaly or peripheral oedema was detected: their LVEFs were more than 20% reduced from baseline. After digitalo-diuretic therapy, they recovered completely during follow-up. All cardiotoxic events were observed during therapy or immediately after the end of treatment, no episode of cardiac function impairment was observed during follow-up.

**Figure 2:**
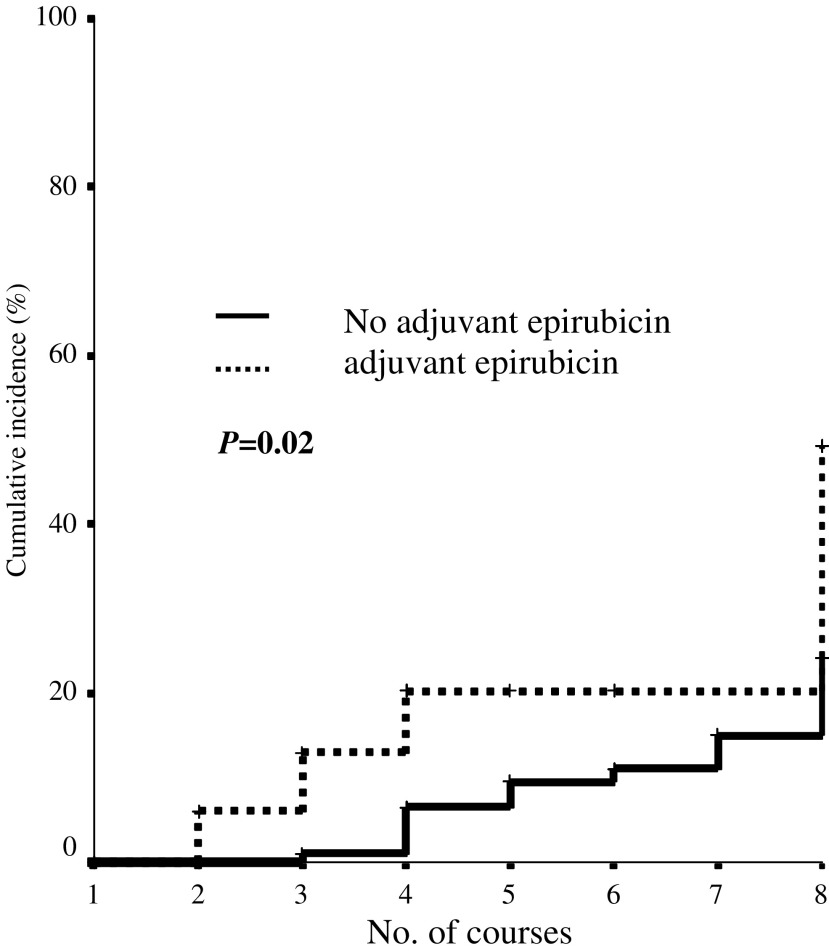
Cardiac risk and cumulative dose of Epirubicin (adjuvant + advanced).

**Figure 3:**
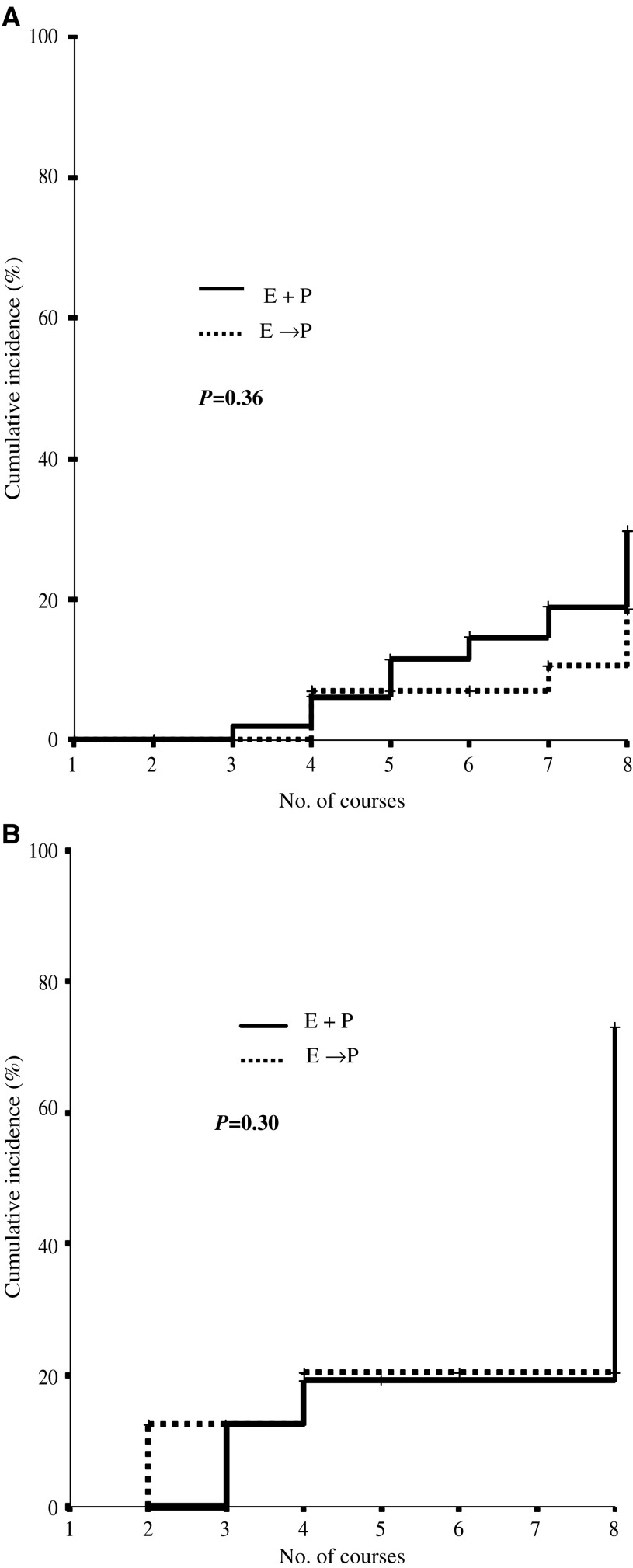
Cardiac risk and randomisation arm: stratification of patients by no prior adjuvant Epirubicin (**A**) and prior adjuvant Epirubicin (**B**).

In the whole population (136 patients), the risk of developing an event was analysed as a function of the cumulative dose of Epirubicin administered (adjuvant treatment plus advanced): the probability of cardiotoxicity was significantly higher in patients receiving prior adjuvant Epirubicin than in patients treated with other adjuvant therapies (*P*=0.02) (Figure 2

). Patients where then stratified according to the type of prior adjuvant treatment (Epirubicin based and free) and the impact of the schedule (concurrent *vs* sequential) was evaluated in both subgroups: no statistically significant impact on the risk of cardiac impairment could be attributed to the schedule and the *P-*values were 0.30 and 0.36 in patients receiving prior adjuvant anthracycline or other adjuvant treatment respectively (Figure 3a and b

).

## DISCUSSION

Anthracyclines (Doxorubicin and Epirubicin) still represent the cornerstone of breast cancer treatment; originally introduced in the metastatic setting, they are now widely used postoperatively in high-risk early breast cancer. Often, in clinical practice, patients treated with adjuvant anthracycline-based combinations, when relapsed, undergo a first-line therapy containing the same drug, if no anthracycline resistance is hypothesised. As a consequence, most breast cancer patients receive high cumulative dose of these drugs during their clinical history. Unfortunately, these molecules cause dose-related cardiotoxicity, mainly consisting in dilated cardiomiopathy, which is irreversible and can be worsened by concomitant treatments or diseases. Cardiotoxicity has become a more important issue in the past decade, with the introduction of taxanes (Paclitaxel first) in clinical practice often combined with anthracyclines. The literature has demonstrated a high rate of CHF when Doxorubicin and Paclitaxel are administered in combination, while the douplet Epirubicin/Paclitaxel can be more safely administered with no excess of cardiotoxicity.

This study reports the results of a prospective evaluation of the cardiac safety of two different schedules of Epirubicin and Paclitaxel. The element that makes the cardiac monitoring we performed different from those published in literature is that, according to the selection criteria, the patients enrolled into this multicenter randomised trial may have received prior Epirubicin-based adjuvant therapy: this subgroup is more and more represented in clinical practice. Considering that 25% of the patients randomised had received prior adjuvant Epirubicin, so as to undergo high cumulative doses of this drug, the percentage of CHFs observed (seven out of 136 patients 5%) as well as the percentage of asymptomatic 20% reduction in LVEF (13.9%) were actually quite low. However, it is important to note that most of the events were registered in patients receiving combination therapy (17 *vs* 9) and all the episodes of CHFs were observed with E+P (7 *vs* 0); as a matter of fact, according to the study design, the planned cumulative dose of Epirubicin is higher in arm A (720 mg m^−2^) than in arm B (480 mg m^−2^). In agreement with this, the cumulative dose of Epirubicin received by the patients (adjuvant therapy plus advanced) is the only statistically significant risk factor for cardiotoxicity emerging from our analysis: this risk is consistent whatever the interval of time between the two strategies, and constantly rises as the number of Epirubicin administrations increase.

After stratification of patients according to the type of adjuvant treatment (anthracycline based and free), we observed that the schedule (combined *vs* sequential) ‘*per se*’ did not significantly affect the risk of cardiotoxicity. However, despite the lack of statistical significance, probably due to the limited number of cardiac events registered, the trends, we observed, are different in the different subpopulations. While in anthracycline-naïve patients the cardiac risk is quite low whatever may be the randomisation arm, according to the published data on the safety of concomitant Epirubicin/Paclitaxel (Conte *et al*, 1997), in patients previously exposed to adjuvant Epirubicin and randomised to receive E+P, the risk of cardiotoxicity gradually increases during chemotherapy, and from 20% after the fourth course, it rises steeply to 75% or more after the eighth course (cumulative dose 1080 mg m^−2^ in some patients). On the contrary, patients randomised to E → P showed a modest increase in cardiotoxicity during the four initial courses of high-dose Epirubicin (up to 21%), but the risk stabilizes, and no additional event was reported during, or after, four courses of high-dose Paclitaxel (250 mg m^−2^). This strongly suggests that taxane, in both arms, has no impact on cardiotoxic events.

The preliminary clinical results of our randomised trial seemed to suggest that the two approaches (concurrent and sequential) were able to give the same benefit (Baldini *et al*, 2002). In terms of activity, E+P provided an earlier opportunity to achieve a complete response: after four courses of therapy, the complete response rates were 4.7 *vs* 1.1 in arm A and B, respectively (*P*=0.22). However, at the end of treatment (eight courses), no difference either in the complete (11.3 *vs* 11%) or overall response rate (58 *vs* 57%) was observed. Overlapping results were also obtained in terms of time to progression and overall survival. While compliance to both strategies was also similar, they significantly differed only for nonhaematologic toxicity, for cardiotoxicity in particular: the risk of CHF and cardiac impairment was higher in the combination arm and especially for Epirubicin pretreated patients. In clinical practice, this data should be taken into account, together with other issues (symptomatic control, quality of life and clinical benefit) when planning a taxane-based strategy in metastatic patients previously exposed to anthracycline. In the light of the equivalence of the two approaches, for this large subgroup, the sequence Epirubicin/Paclitaxel could certainly be a reasonable choice.

## References

[bib1] A’Hern RP, Smith IE, Ebbs SR (1993) Chemotherapy and survival in advanced breast cancer patients: the inclusion of doxorubicin in Cooper type regimens. Br J Cancer 67: 801–805 8471439 10.1038/bjc.1993.146 PMC1968345

[bib2] Baldini E, Salvatori B, Prochilo T, Bolognesi A, Aldrighetti D, Rosso R, Venturini M, Carnino F, Vicentini L, Gallo L, Mammoliti S, Giannessi P, Di Marsico L, Moyano A, Conte PF (2002) Epirubicin followed by paclitaxel *vs* epirubicin plus paclitaxel as first-line therapy in metastatic breast cancer (MBC): preliminary results from a multicenter randomised phase III trial. Proc Am Soc Clin Oncol 21: 52a (abstract 204)

[bib3] Bashtold L, Dalmark M, Susanne BG, Pfeiffer P, Pedersen D, Sandberg E, Kjaer M, Mourisden HT, Rose C, Nielsen OS, Jakobsen P, Bentzen SM (1996) Dose–response relationship of epirubicin in the treatment of post-menopausal patients with metastatic breast cancer: a randomised study of epirubicin at four different dose levels performed by the Danish breast cancer cooperative group. J Clin Oncol 14: 1146–1155 8648369 10.1200/JCO.1996.14.4.1146

[bib4] Cersosimo RJ, Hong WK (1986) Epirubicin: a review of the pharmacology, clinical activity, and adverse affects of an Adryamicin analogue. J Clin Oncol 4: 425–439 3005521 10.1200/JCO.1986.4.3.425

[bib5] Conte PF, Baldini E, Gennari A, Michelotti A, Salvadori B, Tibaldi C, Danesi R, Innocenti F, Gentile A, Dell’Anna F, Biadi O, Mariani M, Del Tacca M (1997) Dose-finding and pharmacokinetics of epirubicin and paclitaxel over 3 hours: a regimen with high activity and low cardiotoxicity in advanced breast cancer. J Clin Oncol 15: 2510–2517 9215819 10.1200/JCO.1997.15.7.2510

[bib6] Criteria Committee of the New York Heart Association (1964) Diseases of the Heart and Blood Vessels. Nomenclature and Criteria for Diagnosis, 6th edn. London United Kingdom: Churchill

[bib7] Danesi R, Innocenti F, Fogli S, Gennari A, Baldini E, Di Paolo A, Salvadori B, Bocci G, Conte PF, Del Tacca M (2002) Pharmacokinetics and pharmacodynamics of combination chemotherapy with paclitaxel and epirubicin in breast cancer patients. Br J Clin Pharmacol 53: 508–518 11994057 10.1046/j.1365-2125.2002.01579.x PMC1874362

[bib8] Early Breast Cancer Trialists’ Collaborative Group (1998) Polychemotherapy for early breast cancer: an overview of the randomized trials. Lancet 352: 930–942 9752815

[bib9] Gehl J, Boesgard M, Paaske T, Jensen B, Dombernowsky P (1996) Combined doxorubicin and paclitaxel in advanced breast cancer: effective and cardiotoxic. Ann Oncol 7: 687–693 8905026 10.1093/oxfordjournals.annonc.a010717

[bib10] Gianni L, Munzone E, Capri G, Fulfaro F, Tarenzi E, Villani F, Spreafico C, Laffranchi A, Caraceni A, Martini C, Stefanelli M, Valagussa P, Bonadonna G (1995a) Paclitaxel by 3 hour infusion in combination with bolus doxorubicin in women with untreated metastatic breast cancer: high antitumor efficacy and cardiac effects in a dose-finding and sequence-finding study. J Clin Oncol 13: 2688–2699 7595726 10.1200/JCO.1995.13.11.2688

[bib11] Gianni L, Munzone E, Capri G, Villani F, Spreafico C, Tarenzi E, Fulfaro F, Caraceni A, Martini C, Laffranchi A (1995b) Paclitaxel in metastatic breast cancer: a trial of two doses by a 3-hour infusion in patients with disease recurrence after prior therapy with anthracyclines. J Natl Cancer Inst 87: 1169–1175 7674322 10.1093/jnci/87.15.1169

[bib12] Norton L (1997) Evolving concepts in the systemic drug therapy of breast cancer. Semin Oncol 24(4) (Suppl 10): 3–10 9275000

[bib13] Perez DJ, Harvey VJ, Robinson BA, Atkinson CH, Dady PJ, Kirk AR, Evans BD, Chapman PJ (1991) A randomised comparison of single-agent doxorubicin and epirubicin as first-line cytotoxic therapy in advanced breast cancer. J Clin Oncol 9: 2148–2152 1960557 10.1200/JCO.1991.9.12.2148

[bib14] Seidman AD, Reichman BS, Crown JPA, Yao TJ, Currie V, Hakes TB, Hudis CA, Gilenski TA, Baselga J, Forsythe P (1995) Paclitaxel as second and subsequent therapy for metastatic breast cancer: activity independent of prior anthracycline response. J Clin Oncol 13: 1152–1159 7537798 10.1200/JCO.1995.13.5.1152

